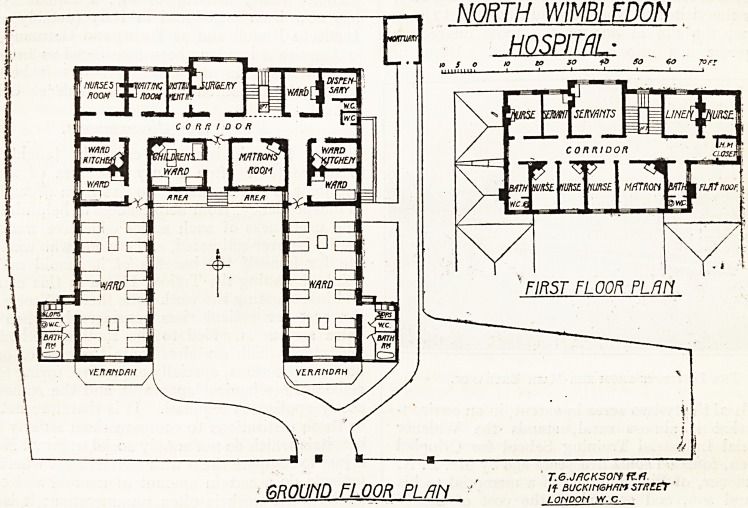# The North Wimbledon Hospital

**Published:** 1912-11-09

**Authors:** 


### HOSPITAL ARCHITECTURE AND CONSTRUCTION.

[Communications on this subject should be marked "Architecture" in the left-hand top corner of the envelope.]

### The North Wimbledon Hospital.

It was only a few weeks ago that we illustrated and described the new Nelson Hospital at Wimbledon. To-day we publish plans of the North Wimbledon Hospital. This building is to take the place of a small cottage hospital built some forty-five or fifty years ago. The site would probably have been sufficient for a really small hospital of the genuine cottage type, but for the building which is now being erected the site is obviously much too small. On three sides the boundary is less than 20 feet from the walls of the hospital.

The plan adopted is one singularly inappropriate to the needs of a hospital. It is, as will be seen, arranged round three sides of a quadrangle, the dimensions of which are about 54 feet by 38 feet. There are two wards, each containing twelve beds, which are arranged in a somewhat novel way in pairs instead of being equally spaced. The sanitary offices are built out at the east and west sides of the wards respectively, and from the position they occupy they must interfere considerably with the light and ventilation of the south end of each ward. The mistake that is so often made has been here repeated, of putting the bath room in the sanitary block, and it is impossible to carry the patient in a reclining position into the bath room, and from the position in which the baths are placed it is impossible to get to both sides of them.

In addition to the large wards there are three wards of one bed each, one of which is placed on the north side, and a small children's ward with apparently four cots in it.

On the north front is a surgery, with instrument room attached.

On the upper floor are bedrooms for the matron, five nurses, and a large room apparently for all the servants.

The architect is Mr. T. G. Jackson, R.A.

## Figures and Tables

**Figure f1:**